# Molecular Mechanisms of Transcription Activation by Juvenile Hormone: A Critical Role for bHLH-PAS and Nuclear Receptor Proteins

**DOI:** 10.3390/insects3010324

**Published:** 2012-03-22

**Authors:** Travis J. Bernardo, Edward B. Dubrovsky

**Affiliations:** 1 Department of Biology, Fordham University, Bronx, NY 10458, USA; E-Mail: tbernardo@fordham.edu; 2 Center for Cancer, Genetic Diseases, and Gene Regulation, Fordham University, Bronx, NY 10458, USA

**Keywords:** *Drosophila melanogaster*, juvenile hormone, nuclear receptor, bHLH-PAS, methoprene-tolerant, germ cell-expressed

## Abstract

Juvenile hormone (JH) is responsible for controlling many biological processes. In several insect species JH has been implicated as a key regulator of developmental timing, preventing the premature onset of metamorphosis during larval growth periods. However, the molecular basis of JH action is not well-understood. In this review, we highlight recent advances which demonstrate the importance of transcription factors from the bHLH-PAS and nuclear receptor families in mediating the response to JH.

## 1. Introduction

The insect life cycle is regulated by two major hormones whose balance determines the course of development. Pulses of 20-hydroxyecdysone (ecdysone) initiate each of the major developmental transitions, including both larval molting and metamorphosis [1], while JH is classically viewed as an antimetamorphic hormone [2]. According to this model, a high titer of JH in the early larval instars directs ecdysone to initiate molting, while the absence of JH during the final instar allows ecdysone to trigger the morphological changes of metamorphosis. The most recent support for this paradigm comes from studies in which manipulating the levels of JH prolongs larval development (increased JH titer) or induces premature metamorphosis (reduced JH titer) [3,4,5]. However, changes in JH titer appear to produce a stronger or weaker effect depending upon the species. In Lepidoptera and Coleoptera exogenously applied JH produces supernumerary larval instars [4,5,6] while in higher Diptera such as *Drosophila melanogaster* the effect of ectopic JH is less dramatic, as it does not prevent pupa formation but inhibits some metamorphic activities, including differentiation of the abdominal histoblasts [7] as well as premature remodeling and apoptosis of the larval fat body [8].

Although much attention has been given to understanding the mechanisms of action for both of these hormones, currently a great deal more is known about signaling by ecdysone than by JH. Ecdysone is a direct regulator of transcription and, like other steroid hormones acts through a nuclear receptor pathway. The ecdysone receptor is heterodimer of the nuclear receptors EcR and USP that binds to the ecdysone response element (EcRE) and activates gene expression when ecdysone is bound to the ligand pocket of EcR [1]. In contrast to the extensive knowledge of ecdysone signaling, the molecular mechanisms underlying JH function remain poorly understood. JH is pleiotropic and controls not only development but also sexual behavior, pheromone production, caste determination, diapause, migration, and the synthesis of female yolk proteins and male accessory gland proteins [9]. In regulating these diverse processes JH appears to utilize multiple pathways, some of which involve the activation of gene expression [10,11,12] while others are transcription-independent [13,14]. The multifunctional nature of JH has impeded the characterization of its signaling mechanisms.

Recently, there has been much progress in understanding the molecular basis of JH signaling. In this short review, we highlight significant advances that have been made in the past two years that demonstrate the importance of transcription factors, including basic-helix-loop-helix Per/Arnt/Sim (bHLH-PAS) and nuclear receptor proteins, in mediating the antimetamorphic activity of JH. We emphasize several unresolved issues, including how JH is integrated into the broader array of signaling pathways and the mechanisms which allow JH to operate distinctly in different insect species.

## 2. MET as a JH Receptor

Methoprene-tolerant (MET) was identified as a potential JH receptor through genetic studies in *Drosophila*, where *Met* null mutants fail to exhibit the morphogenetic defects that result from exogenous application of the JH analog methoprene [15]. MET homologs have since been identified in a wide range of insects, from holometabolans (summarized in [16]) to hemi- and ametabolous species [17]. Given the well-documented ‘status quo’ activity of JH in many insects, it was expected that mutation of the JH receptor would have severe consequences for larval development. This was confirmed in the red flour beetle *Tribolium castaneum*, where removal of *Met* expression produces a premature and lethal initiation of pupation [4]. In *Drosophila*, JH is essential to preadult development as its removal by genetic ablation of the corpora allata results in lethality around the time of head eversion and defects in larval development including precocious apoptosis of the fat body [8,18]. However, *Met* null mutants survive to adulthood and exhibit only minor defects in reproduction and in adult eye development [18,19]. One possible explanation for this paradox is that *Drosophila* possesses an additional *Met-*like gene, *germ cell-expressed* (*gce*) [20], in contrast to insects such as *Tribolium* that have only a single homolog [4]. However, until recently the function of GCE was poorly understood, and in light of the complexity of these observations the role of *Drosophila* MET as a JH receptor has remained controversial.

Studies of JH reception by MET have been scarce, and previously only a single report provided any direct evidence that *Drosophila* MET binds JH [21]. Recently, however, it was found that the ability of *in vitro* translated MET to bind JH is conserved among distantly related insects, including *Drosophila*, *Tribolium*, and the ametabolous *Thermobia domestica* [22]. Importantly, *Tribolium* MET binds JH with nanomolar affinity, an observation that is consistent with the requirement of MET for preventing premature metamorphosis in *Tribolium* [4] and for the induction of JH target genes including the antimetamorphic transcription factor *Krüppel homolog 1* (*Kr-h1*) [23,24]. Taken together, these data strongly indicate that in *Tribolium*, MET functions as a true JH receptor. 

In *Drosophila*, the evidence concerning JH binding is less straightforward. The recent report found that both paralogs are capable of binding JH [22]. However, while MET was shown previously to bind JH with nanomolar affinity [21], these data have not been confirmed in an independent study and it is still not known whether JH is a high-affinity ligand for GCE. A comparative analysis of JH binding affinity for the *Drosophila* paralogs and other insect homologs of MET would help to clear up this issue. The need for a direct comparison is further highlighted by the observation that, despite having a strong affinity for JH [21] *Drosophila* MET was observed to exhibit substantially lower JH binding activity than either GCE or *Tribolium* MET [22]. It is unclear whether the discrepancy results from technical differences associated with the ligand-binding technique employed, but in any case it leaves open the question as to whether MET, GCE, or both proteins function as JH receptors in flies.

Despite the ambiguity of MET and GCE JH binding, several other lines of evidence support the idea that both paralogs do function similarly as JH receptors. First, MET and GCE are both able to mediate JH-dependent gene expression [25,26]. Second, while there are paralog-specific differences in gene structure and in the sequence of some protein motifs [4,27,28] both paralogs exhibit comparable sequence identity to *Tribolium* and other homologs in their PAS domains (Table 1), with most of the divergence between MET and GCE apparently occurring in the C-terminus [28,29]. Finally, genetic studies provide a strong case that both proteins mediate JH signaling *in vivo*. *Gce* null mutants are resistant to exogenous JH and, although fully viable, display reduced fecundity [30]. The *gce* knockout thus phenocopies the *Met* null mutant. Another report found that RNAi-mediated reduction of *gce* expression is lethal, but it was argued that this comes from off-target effects of the dsRNA [19,30]. Nevertheless, in a *Met* null mutant background, overexpressed GCE restores the characteristic methoprene syndrome phenotype [19], and although the capacity for substitution is incomplete–GCE cannot restore oviposition or male sexual behavior in *Met* knockouts [19]–these findings suggest that both paralogs are capable of functioning in JH-dependent processes *in vivo.* Importantly, similar to JH-deficient larvae [8,18] the *Met*/*gce* double knockout mutants fail to undergo head eversion, dying shortly after pupariation, and exhibit premature apoptosis in the larval fat body [30]. These observations demonstrate that MET and GCE are essential to preadult development in *Drosophila* but are functionally replaceable, confirming the belief that the viability of *Met-*deficient flies is attributed to redundancy between the paralogs. Moreover, the genetic studies lend support to the notion that MET and GCE function similarly to their *Tribolium* counterpart in acting as JH receptors *in vivo*. 

**Table 1:** Conservation of *Drosophila* Methoprene-tolerant (MET)/germ cell-expressed (GCE) sequence among insect homologs.

Homolog ^a^	bHLH		PAS A motif		PAS B motif		PAC motif		PAS Domain 1 ^b^		Pas Domain 2	
	MET	GCE	MET	GCE	MET	GCE	MET	GCE	MET	GCE	MET	GCE
DmGCE	82 ^c^	-	71	-	86	-	76	-	69	-	80	-
BmMET_1_	41	41	34	34	65	67	60	63	40	38	62	65
BmMET_2_	62	62	46	38	55	55	51	51	45	45	53	53
AaMET	78	96	71	61	67	76	68	65	69	64	67	69
AgMET	78	92	75	61	72	83	69	66	72	63	70	73
CpMET	74	90	71	63	69	79	66	66	68	64	67	71
TcMET	60	64	55	44	67	72	67	62	49	45	67	67
AmMET	52	54	42	38	60	62	51	51	38	40	55	56
PaMET ^d^	46	53	44	38	44	46	53	53	41	39	49	50
RpMET ^d^	45	45	48	44	48	48	55	51	39	40	52	50
TdMET ^d^	52	55	53	46	60	62	58	56	48	48	59	59

^a^ Dm, *Drosophila melanogaster*; Bm, *Bombyx mori*; Aa, *Aedes aegypti*; Ag, *Anopheles gambiae*; Cp, *Culex pipiens*; Tc, *Tribolium castaneum*; Am, *Apis mellifera*; Pa, *Pyrrhocoris apterus*; Rp, *Rhodnius prolixus*; Td, *Thermobia domestica*; ^b^ Residues comprising the complete PAS domains are described in [29]; ^c^ Numbers indicate percentage sequence identity; ^d^ Truncated bHLH domain lacks the N-terminal basic region.

## 3. JH-Dependent Transcription Mediated by bHLH-PAS and Nuclear Receptor Proteins

Analysis of JH-inducible genes has identified a wide assortment of JH-responsive sequences, suggesting that diverse elements may be involved in mediating or modulating JH-dependent transcription [2]. In general, though, the mechanism of JH-dependent transactivation by these elements is poorly understood. Often the proteins associated with a JH-responsive sequence are unidentified, or else have no known connection to *in vivo* JH function or even a known DNA-binding domain. However, recent efforts to characterize JH target genes have identified transcription factors and cognate response elements that are not only critical to JH-dependent transcription but are also members of the well-known bHLH-PAS or nuclear receptor protein families. 

In *Tribolium* larvae, knockdown of *Met* prevents the expression of several JH-inducible genes, including the *juvenile hormone esterase* gene (*jhe*) and *Krüppel homolog 1* (*Kr-h1*) [23,24]. An RNAi screen targeting other bHLH-PAS genes in *Tribolium* identified steroid receptor coactivator (TcSRC), the beetle homolog of the NCoA/SRC/p160 mammalian family of proteins (hereafter NCoA), as an additional component required for JH-dependent transcription of *jhe* and *Kr-h1* [24]. Two other homologs of TcSRC–mosquito AaFISC and *Drosophila* Taiman–can also mediate the JH response. Taiman, AaFISC, and TcSRC each interact JH-dependently with MET through their PAS domains [22,24,25]. All three heterodimers are able to activate transcription by binding to a JH-responsive sequence identified upstream from the mosquito *early trypsin* gene. Not surprisingly, this JH response element (JHRE) contains an asymmetric E-box-like motif characteristic for the bHLH-PAS transcription factors [25]. These data suggest a possible mechanism for JH-dependent transcription in which JH stimulates MET to interact with its bHLH-PAS partner Taiman/AaFISC/TcSRC, and this heterodimer then binds to an E-box-like response element in JH target genes. So far the MET/AaFISC heterodimer was detected *in vivo* on the *early trypsin* gene [25], and it will be interesting to see whether other JH targets also contain an E-box-like element bound by a corresponding MET heterodimer. Of particular interest is *Kr-h1*, since it is critical to antimetamorphic action by JH and MET [2,17] and is a JH target gene not only in *Tribolium* [23] but also in *Aedes aegypti* [24,31], *Drosophila* [7,30], *Bombyx mori* [32] and the hemimetabolans *Pyrrhocoris apterus* [17] and *Blatella germanica* [33]. 

**Figure 1:**
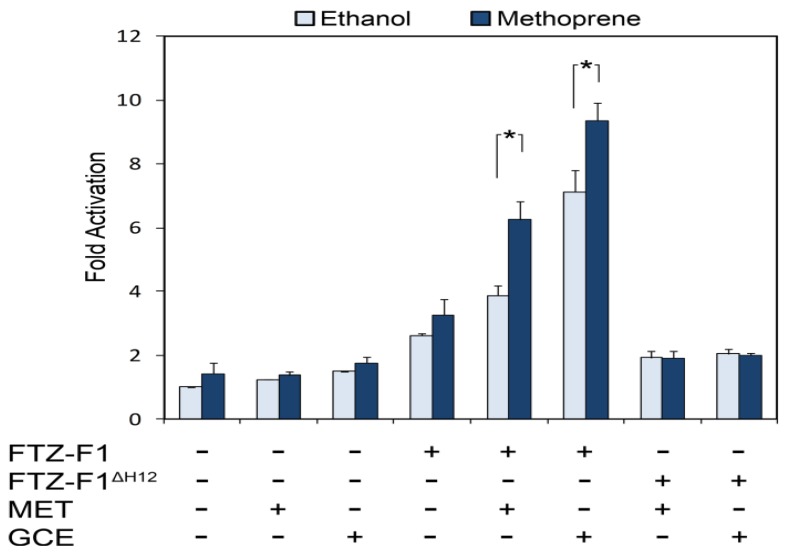
The F1RE is juvenile hormone (JH)-responsive. S2 cells were transfected with expression plasmids for FTZ-F1, MET, or GCE as indicated (x axis) as well as reporter containing ten copies of the FTZ-F1 response element. Luciferase activity normalized to constitutive β-galactosidase activity (y axis) was measured for samples treated with ethanol (light blue) or 5×10^ − 6^ M methoprene (dark blue) for 24 hrs. Asterisk indicates significant (p < 0.05) JH-dependent activation. Heterodimer formation is required for activation, as seen by the FTZ-F1^ΔH12^ mutant which interacts poorly with MET and GCE [29]. Data are shown as the mean ± S.D. from three independent experiments.

Another JH target, the *Drosophila E75A* gene, is in fact under dual hormonal regulation and can be activated by either ecdysone or JH [34]. Both responses are primary, as they do not require concurrent protein synthesis. Ecdysone activation is mediated by the EcR/USP heterodimer which binds to multiple EcREs distributed throughout the 30 kb region upstream from the *E75A* transcription start site [35]. JH activation of this gene requires GCE but appears not to require MET, representing the first example of endogenous JH-dependent transcription mediated by GCE [26]. An RNAi screen against nuclear receptors found that JH activation of *E75A* also requires an orphan receptor, FTZ-F1. Interestingly, the FTZ-F1 response element (F1RE) confers JH induction, as both MET and GCE can activate transcription of a reporter downstream of a DNA sequence containing F1REs (Figure 1). However, the precise role of FTZ-F1 in JH signaling is not understood, and it is unclear whether the F1RE is sufficient to mediate endogenous JH-dependent transcription. While heterodimerization with FTZ-F1 is clearly JH-dependent [29], MET and GCE mediate only weak JH-dependent transcriptional activation through the F1RE, suggesting that other proteins or DNA elements may also be involved in the JH response. Indeed, this possibility is consistent with the role of FTZ-F1 in ecdysone activation: in mosquitoes, FTZ-F1 facilitates ecdysone-dependent transcription by reinforcing DNA-binding of the ecdysone receptor to an ecdysone target gene containing both EcRE and F1RE sequences [36]. A similar mechanism may be at work for JH induction, whereby the F1RE operates in conjunction with other motifs such as the E-box-like response element. The identification of *in vivo* FTZ-F1 binding sites at several *E75A* enhancers [26] may eventually lead to additional sequences involved in JH-dependent transcription.

## 4. Structural Elements of MET Involved in JH Reception and Function

Consistent with its role as a JH receptor, MET possesses structures commonly involved in transcriptional regulation, including a bHLH and two PAS domains in its N-terminal half. The bHLH is a well-studied domain involved in partner dimerization and direct contact with DNA [36]. It is highly conserved among MET homologs [27,28] and adopts the classic helix-loop-helix conformation [29], a clear indication that this domain likely functions in a manner similar to other bHLH-PAS transcription factors. In contrast to bHLH, the structure of other regions of MET has until recently been poorly characterized. Several studies now provide additional information about the structure of MET, revealing features that are critical to its JH-dependent activity and establishing MET as a hormone receptor. 

### 4.1. Structure and Function of the PAS Domains.

MET and GCE possess two conserved PAS motifs, PAS A and PAS B, that are a hallmark feature of bHLH-PAS proteins. PAS domains typically mediate protein-protein interactions, and for bHLH-PAS proteins participate in partner dimerization [37]. Deletions within either of the PAS motifs of MET disrupts dimerization with bHLH-PAS partners [25,38], indicating that both PAS domains are critical to the regulatory actions of MET. 

Using a homology modeling approach [29], MET and GCE were shown to adopt two tandem canonical PAS folds, with a PAS motif comprising the N-terminal half of each fold. The C-terminal half of the second PAS fold is composed of a PAS-associated C-terminal (PAC) motif, as is often the case for PAS domains [39], while the C-terminal half of the first PAS fold is composed of discrete conserved sequences located between the PAS A and PAS B motifs. These sequences form secondary structural elements of the PAS fold in a manner analogous to the elements in a PAC motif, but they are separated by disordered loops whose presence significantly lengthens the region comprising the first PAS domain [29]. While the structure of the PAS domains awaits empirical determination, the models should aid future efforts to understand the molecular basis of bHLH-PAS partner formation by MET. Although a few critical residues have been identified in this respect [22,38], a more comprehensive analysis has not been undertaken.

The presence of loops within the first PAS domain is intriguing, as loops are often essential to PAS-dependent signaling. For example, the *Neurospora crassa* photoreceptor Vivid is a small PAS protein whose flexible loop participates in binding the essential light-sensitive cofactor flavin [40]. Interestingly, the length and sequence of the PAS loops are highly variable among the insect homologs of MET [29], suggesting that they may provide functional diversity to this family of proteins. However, the precise role of these loops awaits further study.

**Figure 2:**
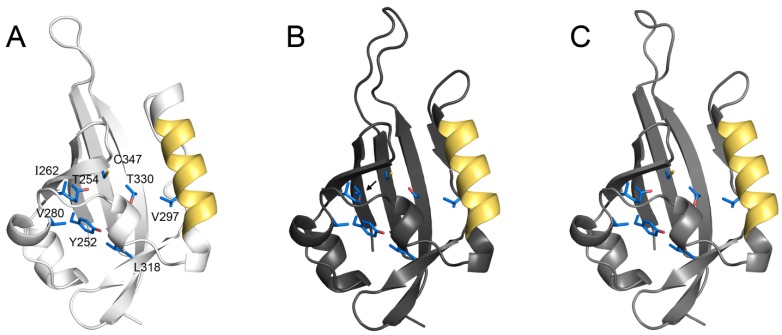
Conserved structure of the MET ligand binding domain. Homology models of the second PAS domain for *Tribolium castaneum* MET (**A**) and *Drosophila melanogaster* GCE (**B**) and MET (**C**) were generated using HIF2α (PDB ID 3F1P), showing that the overall domain structure is conserved. Residues shown above were implicated in JH binding for *Tribolium* MET [22] and are identical in *Drosophila* MET and GCE, except for a Ser substitution in GCE (arrow). An α-helix containing NES-2 is shown in gold.

PAS domains can function as signal sensors for a wide array of compounds [41], and their presence in MET supported the initial idea that MET might be a JH receptor. Indeed, the second PAS domain of *Tribolium* MET appears to be necessary and sufficient for JH binding, for JH-sensitive MET homodimerization, and for heterodimerization with the bHLH-PAS partner Taiman/AaFISC/TcSRC [22]. Modeling of JH bound to this domain revealed several critical hydrophobic residues which, when mutated, block each of these JH-dependent events, leading to the suggestion that MET uses its second PAS domain as a ligand binding domain (LBD) through which JH stimulates a conformational change from an inactive MET homodimer to an active heterodimer [22]. A critical test of this hypothesis would involve a comparison of empirically-derived LBD structures in the JH-bound and unbound states. Moreover, while comparative homology models (Figure 2) provide some support for the idea that *Drosophila* MET and GCE can bind JH in a manner similar to *Tribolium* MET, empirical structures would certainly help to resolve whether both *Drosophila* paralogs function as JH receptors.

### 4.2. Role of JH in MET Nuclear Localization.

The ability to direct proteins into subcellular compartments is critical to cell function. For ligand-dependent transcription factors, translocation to and from the nucleus is often a dynamic process involving multiple nuclear localization (NLS) and export (NES) signals. Ligand often modifies the strength of these signals, thus influencing gene expression by determining the subcellular location of a protein [42]. The import/export signals in *Drosophila* MET were recently identified using ectopically expressed, YFP/CFP-tagged protein in mammalian cells [43]. MET possesses import and export motifs following the bHLH domain (NLS-1 and NES-1), within the second PAS domain (NLS-2 and NES-2), and an unidentified NES in the C-terminus. The presence of multiple NLS/NES motifs suggests that the localization of MET could be regulated dynamically, similar to other hormone-dependent transcription factors. However, *Drosophila* MET is exclusively nuclear both in cell culture [21,43] and *in vivo* [44]. This appears to result from the presence of NLS-1, which exhibits dominance over other motifs and forces JH-independent nuclear localization. In its absence, NES-2 in the second PAS domain causes MET to accumulate in the cytoplasm and enter the nucleus only in the presence of JH [43]. While the mechanism of JH-dependent nuclear entry is unclear, it is noteworthy that the α-helix containing NES-2 participates in ligand binding by providing at least one critical residue [22] to the pocket (Figure 2). This suggests that JH may alter the activity of import/export signals in the second PAS domain by direct interference or by inducing a conformational change in the protein. Interestingly, the dominant NLS-1 of *Drosophila* MET is absent from other homologs, including GCE. This indicates that in *Drosophila*, MET has evolved as an exclusively nuclear protein while JH may play an important role in directing GCE to the nucleus. However, the extent to which JH-dependent subcellular localization occurs in GCE and in other insect homologs remains to be determined.

### 4.3. NR Box in MET C-Terminus Enables JH-Dependent Interaction with Nuclear Receptor FTZ-F1

While some bHLH-PAS proteins function as DNA-binding transcriptional activators, others operate through an entirely separate mechanism. bHLH-PAS proteins from the NCoA family do not dimerize through PAS-PAS interactions and lack the ability to bind DNA [45]. They instead serve as transcriptional coactivators, interacting with a nuclear receptor partner using a centrally located LxxLL (NR box) motif [46]. The NR box is an α-helical structure that binds to activation function 2 (AF2), a hydrophobic binding groove in the LBD of nuclear receptors [47]. NCoA proteins typically possess several NR boxes, with each used to interact with a different group of nuclear receptors [46]. MET and GCE were recently found to possess a conserved motif C-terminal to the second PAS domain–LIxxL–that enables them to interact with the nuclear receptor FTZ-F1 [29]. This motif appears to function as a non-conventional NR box, as modeling of this motif in complex with FTZ-F1 shows a typical NR box/AF2 interaction with some mechanistic differences, such as the dispensability of the canonical charge clamp residues frequently used by nuclear receptors [47]. Similar to the NCoA proteins, MET thus appears to utilize an NR box to participate in signaling pathways through nuclear receptor interaction. 

A crucial feature of MET that distinguishes it from the NCoA family, however, is that it acts as a ligand-binding protein. An important question therefore remains: what is the role of JH in regulating the activity of the MET NR box? For NCoA proteins, the NR box-containing region is sufficient for the interaction with nuclear receptors [48,49,50]. Moreover, the hormone-dependence of NCoA/nuclear receptor interactions is attributed to ligand binding by the nuclear receptor–constitutively active steroid receptors, including mammalian homologs of FTZ-F1, can bind NCoA proteins independently of hormone [51,52,53,54]. In contrast, the interactions of MET and GCE with FTZ-F1 are substantially enhanced by JH [29] and deletions in the second PAS domain disrupt the interaction with FTZ-F1 even when the NR box is intact (unpublished observations). This indicates that the NR box may only be functional when MET is in an active, JH-bound conformation. 

**Scheme 1:**
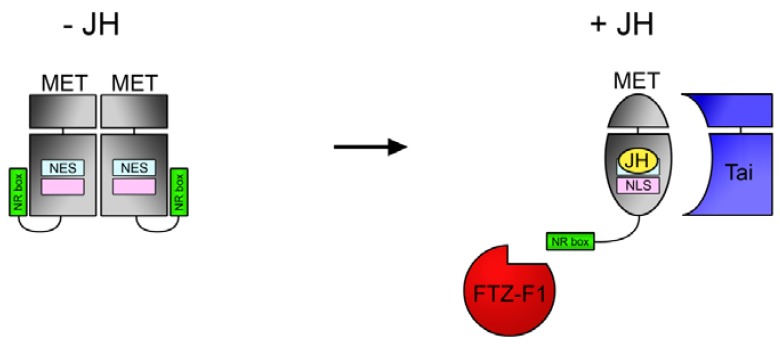
Model of MET activation through JH-dependent conformational changes. In the absence of JH (left), MET exists as a homodimer through interactions in the bHLH and PAS domains (gray). Binding of JH (right) stimulates an active conformation, promoting interaction with Taiman/AaFISC/TcSRC using the bHLH-PAS region, and with FTZ-F1 using a C-terminal NR box. JH may also influence the activity of nuclear export (NES) and import (NLS) signals in the ligand binding domain, promoting nuclear localization. Model is based primarily on evidence from [22,29,43].

Based on the recent studies of MET structure and function [22,29,43], we suggest a model for the role of JH in the regulatory activity of MET (Scheme 1). Binding of JH within the PAS domain triggers conformational changes that break the MET homodimer and promote important JH-dependent events including interaction with bHLH-PAS proteins, interaction with nuclear receptors, and possibly nuclear localization. With its dependence on ligand in mediating interactions with both bHLH-PAS and nuclear receptor partners, MET thus occupies a unique position among the hormone receptors.

## 5. Crosstalk between JH and Ecdysone

Although the molecular mechanisms underlying JH action are gradually being elucidated, a critical question remains: what is the role of JH in the broader context of insect development? This question has grown increasingly complex with the observation that JH signaling involves more than one pathway [13,14]. Moreover, Jones recently demonstrated [55,56] there are at least natural farnesoid hormones–methyl farnesoate, methyl epoxyfarnesoate (JH III), and methyl bisepoxyfarnesoate–involved in larval maturation and metamorphosis. An added complexity is the interplay between JH and other developmental signals, as JH interacts with the Target of rapamycin (TOR), Wingless (Wg), and TGF-β pathways [57,58,59]. One of the most prominent examples of crosstalk involves the ecdysone signaling pathway: JH is thought to play a critical role in preadult development by altering the developmental outcome of early larval ecdysone pulses. While it is still not understood how these two hormones might interact during development, the recent identification of transcription factors involved in the JH response has provided some clues regarding possible mechanisms of JH/ecdysone crosstalk.

Expression of *Kr-h1* is essential to preventing precocious metamorphosis in the early larval development of both holometabolous and hemimetabolous insects [17]. The JH/MET signaling pathway that activates *Kr-h1* is thus an evolutionarily ancient strategy used to regulate the timing of larval development. In holometabolous insects, who transition to adulthood through a pupal stage, this pathway accomplishes its antimetamorphic function by repressing the expression of *broad* [17]. The *broad* gene is an ecdysone target activated at the onset of metamorphosis and is a critical pupal specifier: in *Drosophila*, misexpression of *broad* prematurely commits larvae to pupal development, while in its absence larvae develop normally but cannot pupariate [60]. Direct evidence for JH-dependent suppression of *broad* has been observed in *Tribolium*: removal of *Met* or *Kr-h1* results in precocious expression of *broad* accompanying precocious metamorphosis [23,61], while exogenous JH suppresses *broad* and thereby stimulates a supernumerary larval molt [62]. Until recently, it was unclear whether JH had a similar effect on *broad* expression in *Drosophila*. It has now been demonstrated in the larval fat body that genetic ablation of the CA or double knockout of *Met* and *gce* results in reduced *Kr-h1* expression and precocious expression of *broad* concomitant with precocious apoptosis [30]. These findings suggest that at least in some larval tissues suppression of *broad* by the JH signaling pathway is critical to the timing of preadult development. It remains to be seen whether this phenomenon is a general feature of larval tissues.

In addition to repressing the ecdysone target *broad*, MET may also mediate crosstalk by physically interacting with components of the ecdysone signaling pathway. There have been several documented cases in which JH directly modifies the transcriptional response to ecdysone [63,64,65] and new evidence suggests that interactions between JH and ecdysone signaling proteins may play an important role in preadult development. Consistent with its homology to the NCoA family, Taiman/AaFISC/TcSRC functions not only as a JH-dependent interacting partner of MET [22,24,25] but also as an ecdysone receptor coactivator: it is critical to ecdysone-dependent regulatory processes *in vivo* and facilitates transcriptional activation by ecdysone [36,66,67]. Based on this observation, a model for crosstalk has been suggested [24,25] in which the use of Taiman/AaFISC/TcSRC by both hormones allows JH to antagonize ecdysone-dependent transcription by recruiting the shared protein to JH target genes. Antagonism through the use of shared transcription factors is a common mechanism of crosstalk between nuclear receptors as well as between nuclear receptors and bHLH-PAS proteins [68]. Ecdysone and JH could therefore coordinate their responses by competing for proteins involved in both signaling pathways, such as Taiman/AaFISC/TcSRC or FTZ-F1. The case of FTZ-F1 is particularly interesting in light of the observation that, during larval development, FTZ-F1 is briefly expressed prior to ecdysone pulses in the first two instars [69]. Since FTZ-F1 promotes metamorphosis by facilitating activation of the ecdysone genetic hierarchy [70], an active JH signaling pathway in larvae might prevent precocious metamorphosis in part by competing away FTZ-F1 from the ecdysone pathway. However, there is still no direct evidence that proteins involved in both ecdysone and JH signaling pathways contribute to crosstalk *in vivo.*

## 6. Conclusions

Significant advances have been made with respect to understanding the molecular mechanisms governing JH action. The evidence so far suggests that JH mediates its antimetamorphic function at the transcriptional level. By acting as a ligand for the bHLH-PAS transcription factor MET, JH stimulates an interaction between MET and partner proteins from the bHLH-PAS and nuclear receptor families, allowing MET to activate JH target genes containing E-box-like or FTZ-F1 response elements. Moreover, the involvement of 20E signaling components in the JH pathway could have implications for hormonal crosstalk. While it appears that both MET and GCE function as JH receptors, more detailed analyses of their JH binding, partner dimerization, nuclear localization, and transcriptional activation will illuminate the role of the JH signaling pathway in directing insect development.
